# *vcfsim*: flexible simulation of all-sites VCFs with missing data

**DOI:** 10.1186/s12859-026-06453-9

**Published:** 2026-04-28

**Authors:** Paimon Goulart, Kieran Samuk

**Affiliations:** 1 Department of Computer Science and Engineering, The University of California, Riverside, Riverside, CA USA; 2 Department of Evolution, Ecology, and Organismal Biology, The University of California, Riverside, Riverside, CA USA

**Keywords:** Software, Variant Call Format (VCF), Simulation, Coalescent, Population genetics, Missing data, Ploidy, Demography, Benchmarking, Genomic data analysis

## Abstract

**Background:**

VCFs are the most widely used data format for encoding genetic variation. By design, standard VCFs do not include data from sites where all individuals are homozygous for the reference allele (“invariant sites”) and thus do not differentiate these from sites where data are completely missing. However, missing data are a key feature of biological datasets across all domains of genomics, and many recent studies have shown that missing data can introduce a variety of statistical biases in the estimation of key population genetic parameters. A solution to this limitation is to include invariant sites in a standard VCF, creating an “all-sites VCF”, exposing missing and invariant sites explicitly. One hurdle to the wider adoption of all-sites VCFs is a reliable parameterized simulation framework for generating biologically realistic all-sites VCFs.

**Results:**

Here, we introduce an open-source command-line tool, *vcfsim*, that interfaces with the popular coalescent simulation platform *msprime* and provides convenience functions for simulating all-sites VCFs with variable levels of ploidy and missing data. We show that the post-processed VCFs generated using *vcfsim* align precisely with population genetic expectations (i.e. are statistically identical to raw *msprime* output), accurately introduce missing data in a variety of patterns, and permit the simulation of data with varying ploidy levels, including the simulation of intraindividual ploidy variation (e.g. heterogametic sex chromosomes) and population structures.

**Conclusions:**

Our results demonstrate *vcfsim* is a useful and easy-to-use tool for the benchmarking of new software tools, performing population genetic inference, training of machine learning models, and the exploration of the effects of missing data in genomics datasets.

## Background

The Variant Call Format (VCF) is the most widely used file format for encoding genetic variation data, particularly in the context of population genetics and genomics studies [1]. VCF files are designed to store information about genetic variants, including single nucleotide polymorphisms (SNPs), insertions, deletions, and structural variants. Despite their widespread usage, VCFs have several key limitations. One significant drawback is that, by design, sites where all individuals are homozygous for the reference allele, i.e. invariant sites, are typically omitted [1]. This feature makes it impossible to distinguish between sites that are missing due to lack of sequencing data and sites that are simply invariant. This missing information complicates downstream analyses, particularly in cases where distinguishing between these types of sites is crucial, such as the estimation of nucleotide diversity within and between populations [2, 3].

One extremely common workaround is the use of "all-sites" VCFs, which include both variant and invariant sites. By explicitly including invariant sites, missing data are exposed—missing sites are truly missing and not either missing or invariant. All popular variant calling pipelines include the ability to produce all-sites VCFs (e.g. [4, 5]). Popular tools such as *pixy* [2] and piawka [6] make use of all-sites VCFs to calculate population genetics statistics while accounting for missing data and per-site differences in sample size. However, benchmarking tools that make use of all-sites VCFs has been challenging due to the lack of native support from common simulation packages, which typically output standard (i.e. variants only) VCFs. Simulating all-sites VCFs typically involves complex and ad-hoc workflows, especially when missing data and other biological details are involved.

Indeed, missing data are widely known to introduce serious biases in the estimation of a variety of population genetic parameters [2, 3, 7, 8], yet there are no widely adopted tools for simulating ground-truth data with predefined levels of missing genotypes or sites. This is an issue, as researchers often need simulated data with specific patterns of missing information or population genetic parameters to validate tools or analyses. However, current methods require ad-hoc solutions and lack flexibility. As such, there is a need for standardized tools to simulate population genetic data and output all-sites VCFs, both for working population genetics researchers and bioinformaticians developing new tools.

Here, we introduce *vcfsim*, a command-line tool that provides a user-friendly interface that produces all-sites VCFs using the coalescent simulation package *msprime* [9] with customizable levels of missing data. The tool also supports simulation of VCFs across various ploidy levels, including mixed ploidies (e.g., diploid organisms with haploid sex chromosomes), as well as simulation of multiple evolving populations. This makes *vcfsim* a flexible and accurate tool for rapidly simulating genetic data. By addressing the limitations of current tools, *vcfsim* simplifies the process of creating biologically realistic VCFs for use in testing and validating genetic analysis pipelines, as well as in inferential tasks needing large amounts of simulated data such as approximate Bayesian computation.

## Implementation

**Fig. 1:**
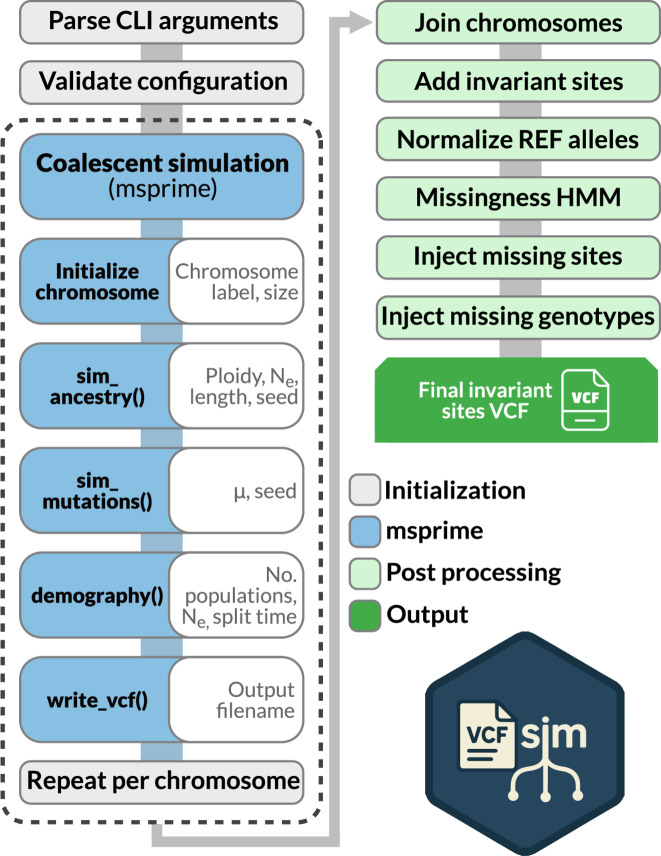
Flow chart showing the basic operation of *vcfsim*. Each step in the software (bubbles) is coded by its general function (see inset legend). The general flow of data through the program is indicated by the dark gray/blue line behind the steps. The coalescent simulation portion (dashed outlined) is repeated for each chromosome in multichromosome mode. The *msprime* simulation steps have user specified parameters (white interior bubbles) parsed from the command line invocation of *vcfsim*

We developed *vcfsim* in Python version 3.11 leveraging tools from the following libraries: ipython [10], numpy [11], and pandas [12]. The coalescent simulation package msprime [9] provides the core population genetic-based backend for the simulation. The source code for *vcfsim* can be found at http://github.com/samuk-lab/vcfsim, and is freely available via MIT license. The general flow of the software is depicted in Fig. 1. It can also be easily installed via the package management platform conda [13], on bioconda [14]: https://bioconda.github.io/recipes/vcfsim/README.html.

*vcfsim* is a VCF simulator that combines *msprime*-based ancestry and mutation simulation with explicit, VCF-aware post-processing (Fig. 1). The program first parses command-line arguments and validates the configuration, then iterates over chromosomes, initializing chromosome metadata and simulating genealogies, mutations, and user-specified demographic histories using *msprime*. For each chromosome, the resulting variants are written to an all-sites VCF that includes invariant positions. After simulation, *vcfsim* performs a series of post-processing steps: chromosomes can be joined, reference alleles normalized, and missing data introduced in a controlled manner. Missingness is injected both at the site and genotype level, optionally using a hidden Markov model to generate realistic spatial autocorrelation in missing data. The final output is a standards-compliant VCF with invariant sites and empirically realistic patterns of variation and missingness, suitable for benchmarking population-genetic methods and pipelines.

*vcfsim* simulates realistic genetic variation data that can be highly customized to match a wide variety of use cases. Our focus was on an easy to deploy, one-step solution for simulating VCFs for all kinds of biological applications. Unlike tools such as *vcfgl* [15], our tool was designed for hard-filtered VCF workflows (i.e. treating all genotypes that pass filters as completely known, and not operating on genotype likelihoods), which in our experience are very common. Our tool *vcfsim* works by simulating genetic data from a single population using a neutral coalescent process (via *msprime*), applying mutations to these sequences, outputting the results in “all-sites” VCF format, and post-processing this VCF to simulate missing data of various kinds and variable ploidy. Users can customize the simulation via command-line arguments to specify the desired characteristics of the simulated population and VCF output.

Key features of *vcfsim* include:

*Random seed control*: Users can specify a random seed to control stochastic elements of the coalescent simulation and missing data processes. Identical seeds yield identical results for repeated runs, ensuring reproducibility.

*Missing data simulation*: The software allows for the simulation of missing data, both at the level of genotypes and whole sites. By default, missing sites are introduced with uniform probability according to a user-specified percentage. This results in deterministic and uniformly distributed missingness across all sites. In addition to this method, *vcfsim* allows users to further customize the distribution of missing data by enabling missing data to be spatially clustered via a generative two-state Bernoulli Hidden Markov Model (HMM). Under this model, sites transition between a baseline low-missing state (“low”) and a high-missing state (“high”) according to user-specified transition probabilities. Sites in the high state have a higher probability of being missing compared to sites in the low baseline state. Missingness introduced through the HMM is stochastic, and therefore the exact proportion of missing sites may thus vary between simulations depending on the specified seed. This approach allows users to generate VCFs with spatially clustered missing data, which may offer greater biological realism depending on the user’s objective.

*Flexible chromosome inputs*: Users can simulate multiple chromosomes simultaneously by providing an input file with parameters for each chromosome (e.g. multiple autosomes and a sex chromosome).

*Support for arbitrary ploidy*: Any ploidy level supported by msprime can be simulated, which we extended to handle mixed ploidies such as those found in systems with heterogametic sex chromosomes. This is done using a user-provided chromosome parameter file, in which each row specifies a chromosome label, ploidy, sequence length, effective population size, and mutation rate. Each row of the file (a chromosome) is simulated independently using these parameters, and the resulting simulations are then concatenated into a single multichromosome VCF.

*Multiple populations*: vcfsim can simulate samples drawn from two populations with a shared coalescent history (i.e. derived from a shared ancestor), with varying divergence times and population sizes. By default, *vcfsim* simulates a single population. In the two-population mode, a single ancestral population with Ne = 2Ne splits into two daughter populations of equal effective size at a given divergence time. Each population contains an equal number of samples.

*Efficient handling of large simulations*: *vcfsim* supports running multiple simulation replicates from a single command, facilitating use for tool validation, statistical model fitting, and machine learning model training. It can also be readily parallelized using GNU parallel, for example.

### Parameters and inputs

*Vcfsim* offers a range of user-specified customization options. These parameters include both required and optional arguments that control various aspects of the simulation process, such as the random seed for reproducibility, the level of missing data, and the characteristics of the population being simulated. The required and optional parameters and given in Tables 1 and 2, respectively.

**Table 1:** Required parameters for vcfsim

Parameter	Description
--seed [integer]	The random seed used by *vcfsim* ensures reproducibility. By specifying a seed, users can generate identical VCFs in subsequent runs
--percent_missing_sites [integer]	The percentage of *sites* in the VCF that will be marked as missing. For example, if the value is set to 10, 10% of the VCF sites will be randomly selected to be missing (removed from the VCF entirely). This setting disables the missingness HMM simulation
--percent_missing_genotypes [integer]	The percentage of individual *genotypes* coded as missing VCF for every site. A value of 10% would result in 10% of the samples at each site being coded as missing (e.g. “./.” for diploids per the VCF specification)
--sample_size [integer]	The number of samples to be simulated and included in the VCF output. A sample represents one individual from each simulated population

Each row represents a single required parameter, with its name, value (in capital letters), and type of parameter (e.g. an integer, string, etc.). Square brackets indicate placeholders for actual parameter values. The description column contains a description of the function of each parameter in controlling the behavior of vcfsim

**Table 2:** Optional parameters for vcfsim (i.e. those not required for a run). These parameters extend the basic capabilities of vcfsim

Parameter	Description
--chromosome [string or integer] Default = 1	An arbitrary label for the default case of a single simulated chromosome (see “param_file” below for the case of more than one chromosome)
--replicates [integer] Default = 1	The number of VCF replicates *vcfsim* should produce. Replicates are given incrementally increasing seed values
--sequence_length [integer] Default = 10,000	The length of the genetic sequence being simulated in base pairs
--ploidy [integer] Default = 2	This parameter sets the ploidy level for the simulation (the number of copies of each chromosome per individual)
--Ne [integer] Default = 1,700,000	The effective population size of the simulated population. Along with the mutation rate, this governs the amount of genetic diversity in a neutrally evolving population
--mu [float] Default = 0.0000000055	The per-base mutation rate per generation in the simulated population, influencing genetic diversity among individuals
--output_file [string] Default = myvcftest	The name of the output file where the simulated VCF will be saved. If this argument is not specified, *vcfsim* will print the output to standard out. When multiple replicates are generated, the seed value is appended to the output file name to distinguish between runs
--chromosome_file [string] Default = None	A file name for a plain-text, tab-delimited parameters file where each row contains a unique chromosome name, ploidy, sequence length, effective population size, and mutation rate. The results of each chromosome are concatenated into a single output file
--hmm_baseline [float] Default = None	The baseline probability that a site is missing when the missingness model is in a low-missing state. Lower values result in fewer missing sites in both “low” and “high” regions
--hmm_multiplier [integer] Default = None	A multiplier applied to the baseline missingness probability when the missingness model is in a high-missing state. Larger values increase the probability of missingness in “high” regions
--hmm_p_low_to_high [float] Default = None	The probability of transitioning from a low-missing state to a high-missing state between adjacent sites
--hmm_p_high_to_low [float]	The probability of transitioning from a high-missing state to a low-missing state between adjacent sites
--population_mode [integer]	The number of populations to simulate. By default, a single population is simulated. When set to “2”, vcfsim will simulate a single ancestral population C splitting into two new populations, A and B, at given divergence time (--div_time below)
--div_time [integer]	The divergence time, in generations, used in two population mode
--samples [string]	A space separate list of custom sample names for the samples (individuals) in the resulting VCF. Without this, msprime’s default sample names are used (“tsk1” etc.)
--samples_file [string]	The path to a file containing whitespace separated custom sample names. An alternative to --samples above

Each row represents a single required parameter, with its name, value (in capital letters), and type of parameter (e.g. an integer, string, etc.). Square brackets indicate placeholders for actual parameter values. The description column contains a description of the function of each parameter in controlling the behavior of vcfsim

### Output options

Users can specify an output file for the generated VCF data using the “--output_file” argument. The resulting file will always be in the.vcf format and will also append the random seed used for the simulation at the end of the filename ensuring repeatability of results. The exact command line parameters invoked to produce a specific VCF are also encoded in the “source” metadata field of each VCF. If no output file is specified, the VCF data will be printed directly to standard output. This feature allows users to integrate *vcfsim* easily into command line pipelines, enabling further processing or analysis without the need to generate a static file.

### Validation of accuracy

To assess the biological and numerical accuracy of *vcfsim*, we performed a variety of validation procedures. The accuracy of *msprime* has been thoroughly demonstrated [9]*,* however *vcfsim* performs substantial post-processing and manipulation of VCFs produced by *msprime* via the addition of invariant sites and missing data, multiple chromosomes, and variable ploidy. As such, we sought to validate the following:

First, to evaluate whether the simulated VCFs generated by *vcfsim* align with theoretical expectations of nucleotide diversity (π), we generated 10,000 diploid VCF files, each with a mutation rate of 1e−9, an effective population size (N_e_) of 1,700,000, a sequence length of 10,000 base pairs, and a sample size of 10 (i.e. 10 diploid genotypes). Each simulation was performed with a unique random seed, starting from 1000 and incrementing by 1 up to 11,000. To measure π for each simulated VCF, we employed both VCFtools [1] and *pixy* [2] to ensure consistency between the tools and to verify that *vcfsim* did not introduce any errors. This dual approach also allowed us to cross-check the π values calculated by both VCFtools and pixy.

Similarly, we evaluated whether genetic divergence between populations simulated in two population mode aligned with the theoretical expectation of F_ST_ according to Nei [16] and Hudson et al. [17]. To do this, we repeated the simulation above in two population mode, varying divergence time from 1 to 10,000,000 generations in powers of 10, and ending at a final divergence time of 50,000,000 generations. To estimate mean F_ST_ for each divergence time setting, we used *pixy* to compute Hudson’s F_ST_ between the two populations, and then aggregated the results in R.

To verify that the percent of missing genotypes and missing sites introduced by *vcfsim* align with the specified input values, we obtained an independent validation of the extent of missing data using *bcftools* [5]. For missing genotypes, we generated VCF files with a sample size of 100 and incrementally introduced missing genotypes, ranging from 0 to 100%, increasing by 1% (one missing sample) with each iteration. Similarly, to assess the accuracy of missing site generation, we created another script to manually count the number of missing rows in the VCF files. In this case, we set the sequence length to 100 and progressively introduced missing sites, again in 1% (one site) increments from 0 to 100%. Ideally, we expect a perfect linear relationship between the specified and measured values, confirming that *vcfsim* is accurately applying the desired levels of missing data.

To assess runtime and memory efficiency, we used *vcfsim* to simulate sets of 1000 VCFs with the following parameters: 10 diploid samples, μ = 1e−9, N_e_ = 1.7 M, and sizes of 1000–1,000,000 bp, in steps of powers of 10. We recorded runtime in second and peak memory consumption in megabytes. All tests were performed on an Apple MacBook Pro M2 (8 GB), running macOS Ventura 13.4.1.

We provide several worked examples of the generation of datasets with *vcfsim*. First, we demonstrate variable ploidy: (1) a tetraploid organism, and (2) a diploid organism with two autosomes and a pair of heterogametic sex chromosomes (e.g. similar to a XY human male). In accordance with Wilson Sayres [18], we again computed π, with the expectation of the following levels of average genetic diversity: autosomes π = 4N_e_μ, X chromosome π = 3N_e_μ (¾ of autosomes), and Y chromosome π = N_e_μ (¼ of autosomes). We used the following parameter values for these examples: N_e_ = 1 M_,_ μ = 1e-8, sequence length = 10,000, and sample size = 10.

Secondly, to demonstrate how the missing data HMM can be tuned to control patterns of missing data, we simulated 10,000 bp VCFs using the parameters above with a uniform baseline missingness of 1% with the following HMM settings, where *P*_*LH*_ is the probability of transitioning from the low missing state to the high missing state, and *P*_*HL*_ is the probability of transitioning from the high missing state to the low missing state: disabled (baseline only, *P*_*LH*_ = 0_*,*_* P*_*HL*_ = 0), low clustering (*P*_*LH*_ = 0.05_*,*_* P*_*HL*_ = 0.1), medium clustering (*P*_*LH*_ = 0.005_*,*_* P*_*HL*_ = 0.01), and high clustering (*P*_*LH*_ = 0.0005_*,*_* P*_*HL*_ = 0.001). To illustrate the features of the HMM, we set the high-state multiplier to 100 (i.e. sites in the “high” state have a 1% × 100 = 100% probability of being missing, i.e. always missing), but we provide a parameter to control this. All VCFs were otherwise simulated with identical parameters, and a shared seed of “4000”. We computed the mean and standard deviation of dwell time (time in the high and low states of the HMM), as well as the number and total fraction of runs (unbroken sequential positions with the same state) for each set of HMM parameter settings.

All codes used in the validation procedures and example plots for *vcfsim* are available as a Github repository: https://github.com/samuk-lab/vcfsim-benchmarking-and-test-analysis. All statistical analyses were carried out in R [19], using the tidyverse [20] and vcfR [21] packages.

## Results

### Accuracy

**Fig. 2:**
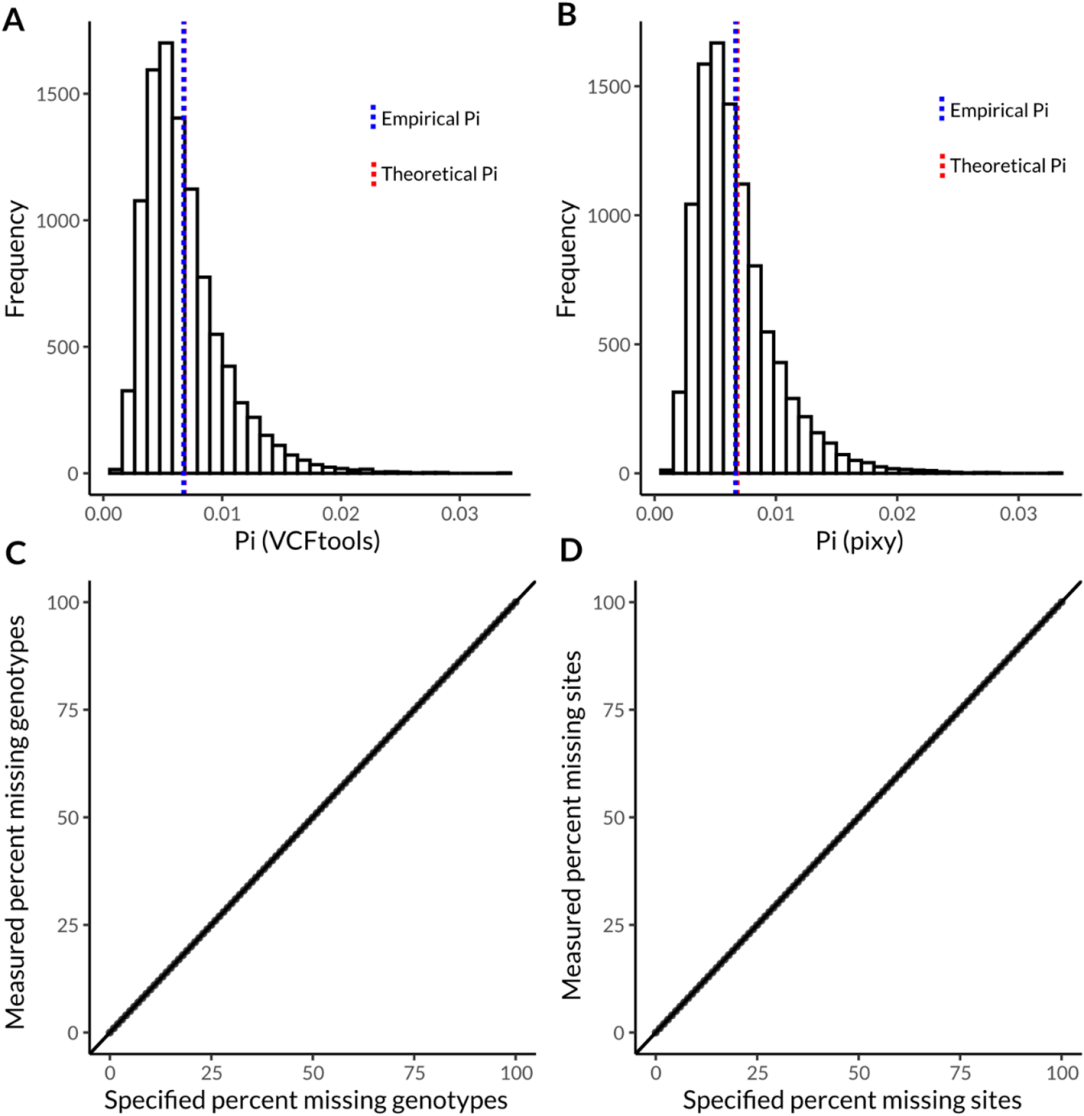
Distribution and accuracy of nucleotide diversity (pi) values and missing data in VCFs produced by *vcfsim*. **a** Distribution of pi values calculated with VCFtools in the absence of missing data. **b** Distribution of pi values calculated with pixy in the absence of missing data. Vertical lines in (**a**) and (**b**) represent the theoretical expectation for pi (red, 4N_e_μ), and the empirical mean of the computed values (blue). **c** Relationship between specified (*vcfsim*) and measured (bcftools) percentages of missing genotypes. **d** Relationship between specified (*vcfsim*) and measured (manual count) percentages of missing sites

The estimated π values calculated from *vcfsim* VCFs using both pixy and VCFtools were identical and closely matched theoretical expectations (Fig. 2A, B; 4Neμ = 0.0068; VCFtools mean π = 0.00676, 95% CI 0.00669–0.00682; pixy mean π = 0.00676, 95% CI 0.00669–0.00682, n = 10,000). We also observed a perfect linear relationship between the amounts of missing data specified by *vcfsim* and the values for missing data measured using *bcftools* and/or the raw count of rows in the VCF (Fig. 2C, D; R^2^ = 1.00, correlation test: *p* < 0.00001, n = 100). This indicates that *vcfsim* correctly implements arbitrary levels of missing genotypes and sites.

**Fig. 3:**
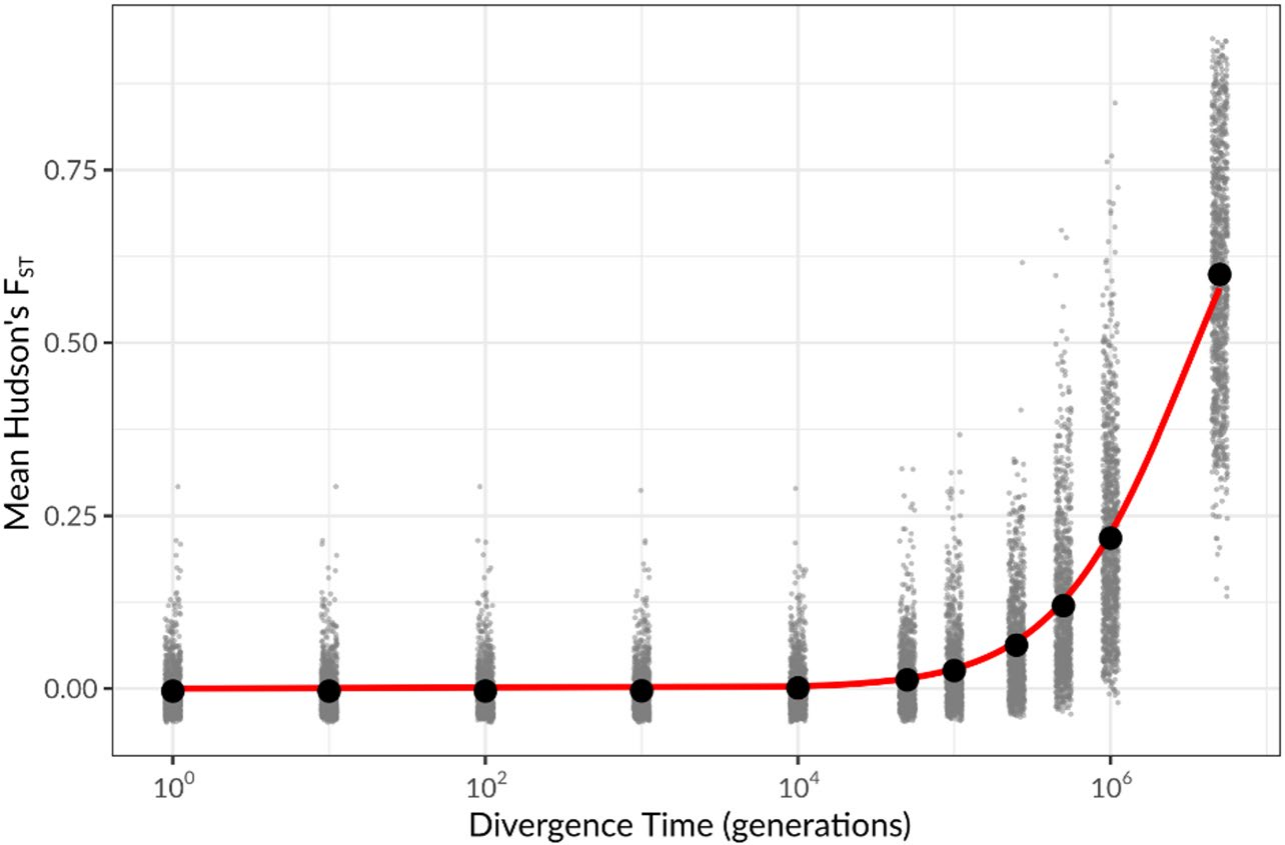
Distribution and accuracy of genetic divergence (Hudson’s F_ST_) simulated across a range of divergence times (log scale) using *vcfsim*. Grey dots represent mean F_ST_ estimates (across all sites in a VCF) from individual simulations at a given divergence time with Ne = 1.7 M, mu = 1 × 10^–8^. Black dots represent the grand mean of all F_ST_ values across replicate simulations for a given divergence value. The red line represents the theoretical expected mean value for F_ST_ under a Jukes-Cantor mutation model [16, 17]

The mean estimated F_ST_ values (Hudson’s F_ST,_ calculated with *pixy*) between populations simulated under the two-population mode exactly matched theoretical expectations of F_ST_ scaling under a Jukes-Cantor mutation model, the default in msprime (Fig. 3, expectations from [16, 17]). The average deviation between the estimated and expected F_ST_ values across divergence times was − 0.002, well within the sampling variance of the simulated values (average 95% CI width = 0.008).

### Runtime and memory consumption

**Fig. 4:**
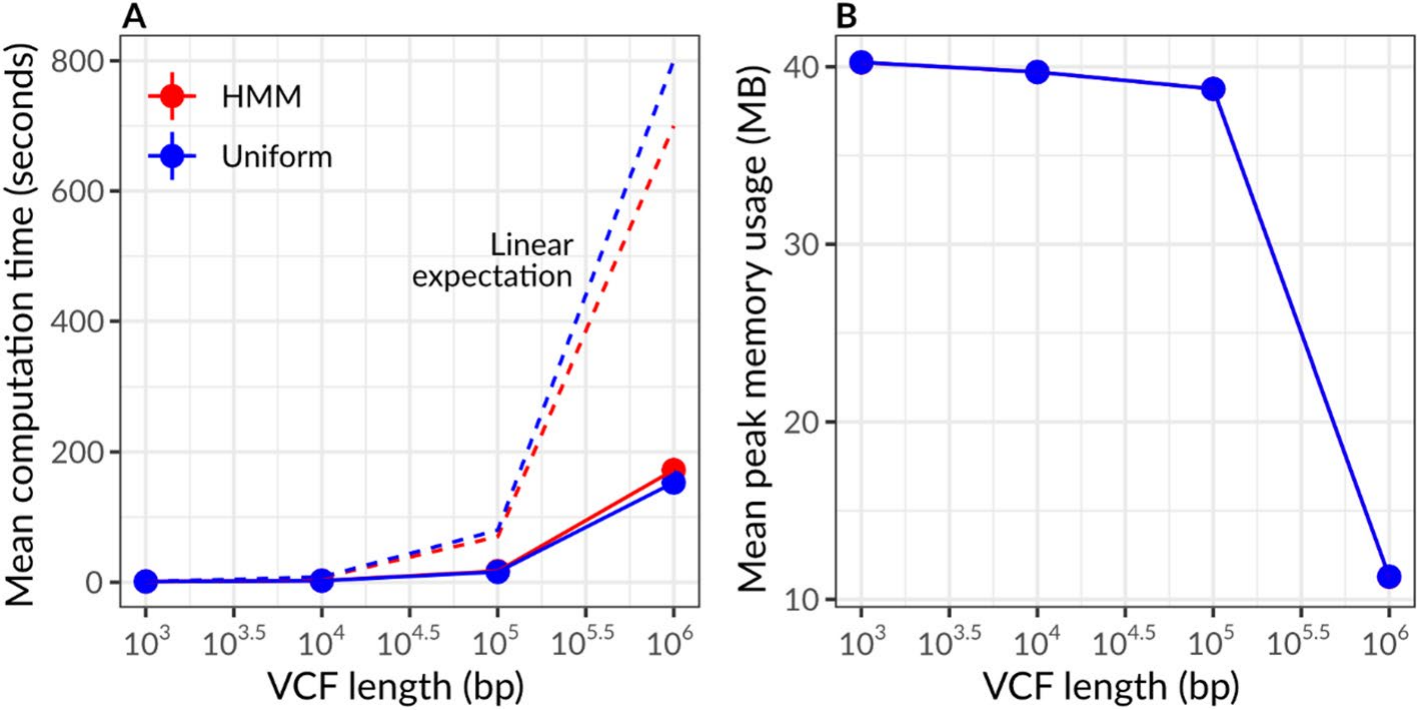
Mean computation time in seconds (**A**) and memory consumption in megabytes (**B**) of VCFs of varying base pair length by *vcfsim.* Each point depicts the mean computation time/memory consumption of 1000 VCFs of the specified length (see “Runtime” Results section for full *vcfsim* parameterization). Dashed lines in “A” represent the expected linear scaling of runtime with VCF length computed by anchoring the relationship at the smallest simulated file size and extrapolating linearly to larger files.VCF length is shown on a log_10_ scale

The runtime to create VCFs (10 diploid samples, mutation rate = 1e−9, Ne = 1.7 M) ranged from an average 0.649 s for a 1kbp VCF to 168 s for a 1Mbp VCF (Fig. 4A, means derived from 1000 replicate simulations). The use of the HMM missing data mode introduced a small overhead (~ 5%) at larger VCF sizes (Fig. 4A, red lines). Using vcfsim, this task shows an approximate scaling relationship of ~ O(N^0.8^) complexity, i.e. efficient and potentially sublinear (Fig. 4A, Best fit power law exponent: T ∝ N^0.815^, 95% CI for exponent k = 0.466, 1.164), where N is the simulated sequence length, T is runtime in seconds, and k is the fitted power law exponent.

Across VCF sizes, memory consumption peaked at around 40 MB and declined as a function of VCF size (Fig. 4B). The decline in memory usage is likely because *vcfsim* generates sites sequentially and streams output to disk, and thus memory usage is dominated by fixed overhead (e.g., the Python runtime, library imports, and per-sample data structures) rather than by the total number of simulated sites. As a result, peak memory usage remained approximately constant across most sequence lengths and in some cases decreased for larger simulations, reflecting differences in I/O buffering, memory reuse by the Python allocator, etc. Importantly, memory usage did not scale with final VCF size, indicating that *vcfsim* does not retain per-site data in memory and instead operates in a streaming fashion.

### Worked examples

**Fig. 5:**
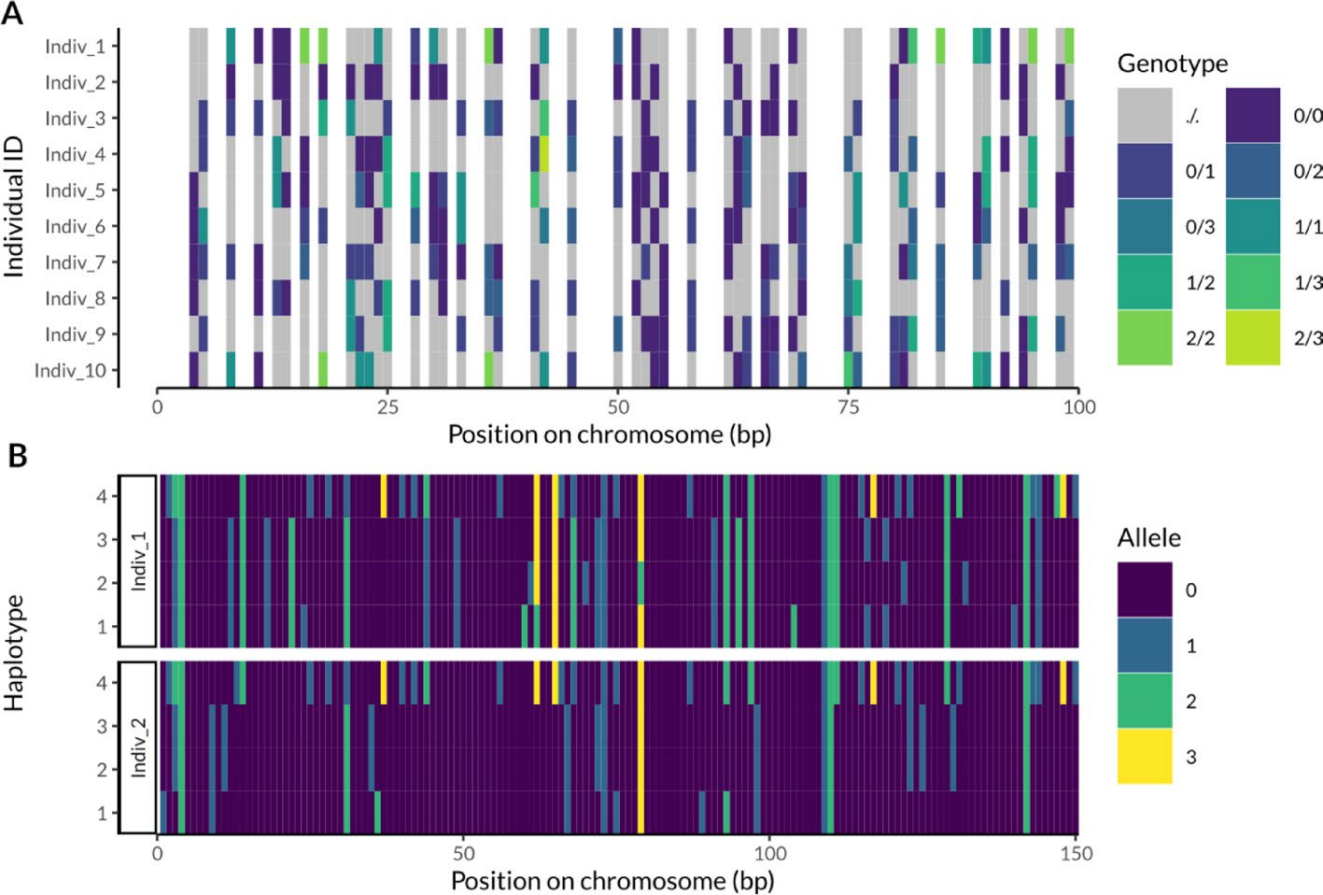
A heatmap visualization of VCF data generated using *vcfsim*. (**A**) A 100 bp span of genetic data from ten diploid individuals simulated using *vcfsim*. Each column represents a site in the genome, and each row a diploid individual. Each cell is colored based on its diploid genotype (note some sites are polyallelic), with grey representing a missing genotype. Completely missing sites are represented as white. (**B**) Simulated VCF of data from two tetraploid individuals with no missing data. Each column represents a site in the genome, and each row one of the individual haplotypes (1–4) for each individual (Indiv_1 and Indiv_2), with cells colored based on the haploid genotype (i.e. allele) at each site. Alleles in both plots are arbitrarily coded 0–4, with zero being the reference allele

**Fig. 6:**
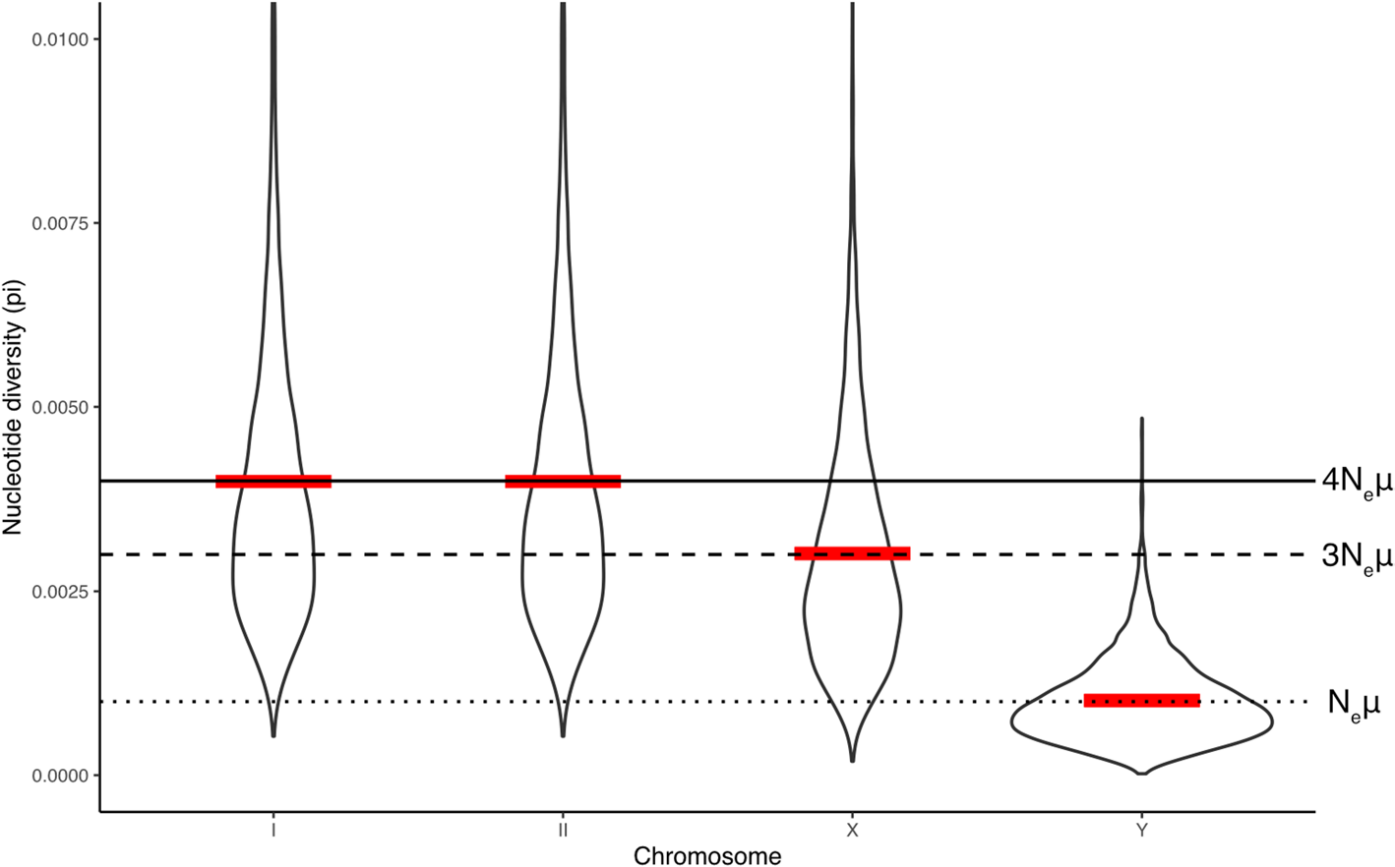
A violin plot of nucleotide diversity (pi) measured in 10,000 bp windows in a diploid individual with autosomes (I, II) and heterogametic sex chromosomes (X, Y). Each violin shows the frequency distribution (density) for nucleotide diversity on each chromosome. Horizontal lines show the theoretical expectation for each class of chromosome (autosomes = 4N_e_μ, X = 3 N_e_μ, Y = N_e_μ). Red lines depict the empirical mean nucleotide diversity for the whole chromosome (expected to match the horizontal black lines)

To showcase additional features in *vcfsim*, we generated representative diploid VCFs displaying missing data (genotypes and sites, Fig. 5A), data from a tetraploid organism (Fig. 5B), as well as data from a diploid organism with heterogametic sex chromosomes (Fig. 6). When multiple ploidy levels are simulated in this manner, the genetic diversity of autosomes and sex chromosomes are consistent with theoretical expectations (N_e_ = 1,000,000, μ = 1e−8; Fig. 6, 4N_e_μ = 0.04, for autosomes, 3N_e_μ = 0.03 for X chromosomes, and N_e_μ = 0.01 for the Y chromosome).

**Fig. 7:**
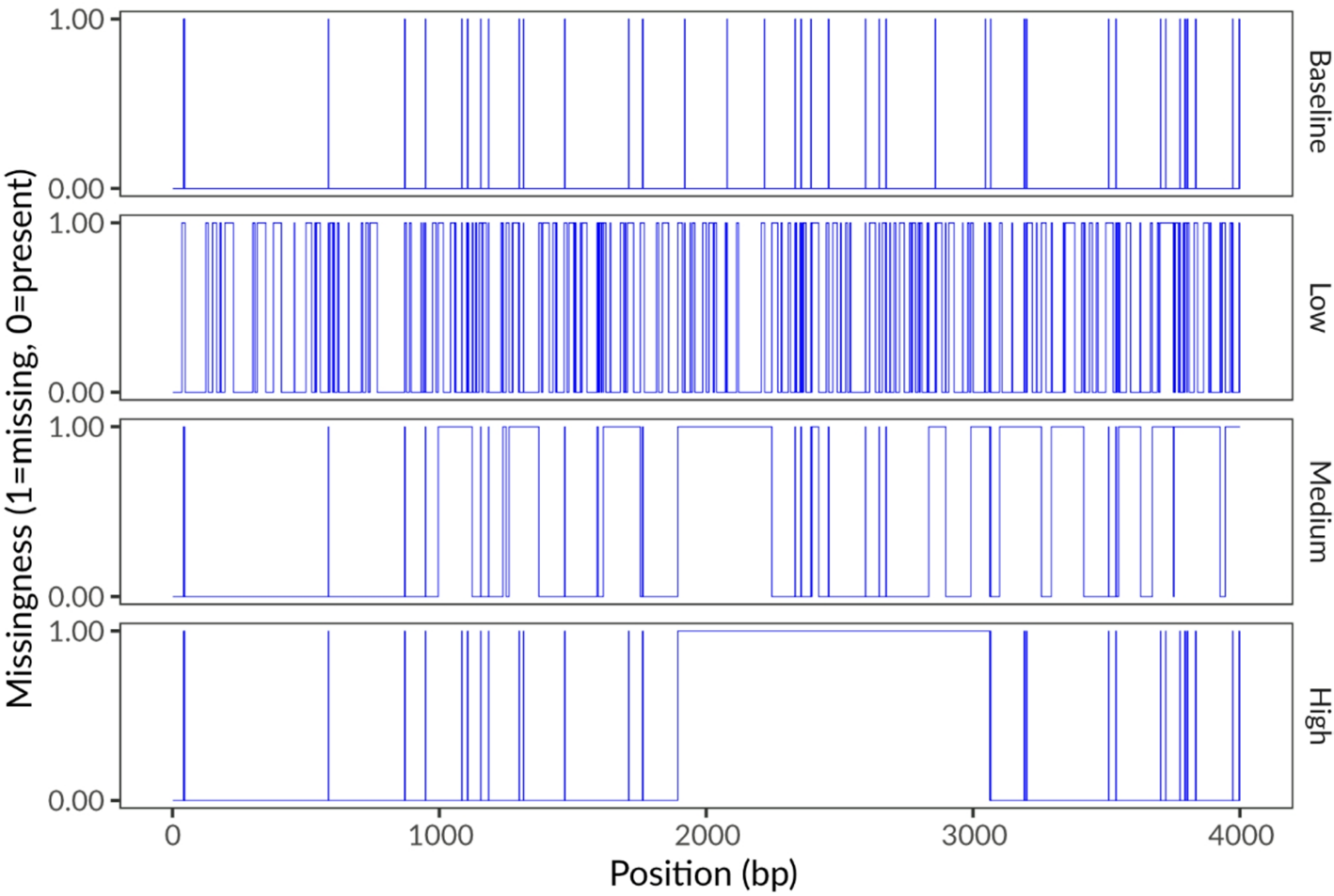
A step plot showing patterns of missingness (site masks) simulated across a range of HMM parameters. Each row depicts the missing state of sites (1 = missing, 0 = present) across a 4000 bp window in a single VCF. “Baseline” represents the uniform missingness model (a fixed per-site probability of missingness), and Low, Medium, and High each represent increasing levels of spatial autocorrelation/clustering of missing data, tuned using the HMM parameters (see text for parameter values). The HMM is layered on top of the baseline uniform model and VCFs were simulated with an identical seed (“4000”), and thus sites missing in the baseline are also always missing in the HMM modes as well

We also provide a visualization and quantification of how the HMM missing data model can be tuned to represent autocorrelation (clustering) of missingness (Fig. 7, Table 3). The model is highly flexible and can represent a wide range of clustering values seen in empirical genomic data.

**Table 3:** Metrics of run length (dwell time, in number of positions), number of runs (count of sets positions in each state), and the total proportion of positions in each state for each of the four HMM parameter settings

HMM Setting	Mean (SD) run length		Number of runs		Prop. positions in state	
	Low	High	Low	High	Low	High
Baseline	105.39 (97.78)	1 (0)	94	93	0.01	0.99
Low	16.58 (16.04)	8.56 (8.73)	398	397	0.34	0.66
Mid	73.51 (83.30)	43.27 (78.65)	86	85	0.37	0.63
High	105.84 (105.02)	26.08 (157.1)	76	75	0.20	0.80

Metrics were calculated for a single run for each setting with a window length of 10,000 bp

## Discussion

*Vcfsim* provides a standardized method for generating all-sites VCFs, complete with configurable levels and genomic patterns of missing data and flexible ploidy and chromosome configurations. It provides a simple and customizable interface to the widely used coalescent simulator msprime [9], along with parameterized postprocessing of VCFs to add invariant and missing sites. The ability to customize these parameters allows for the creation of test datasets that can model key aspects of real-world population genetic datasets, making it a useful tool for a variety of applications in genomics and population genetics.

We demonstrate that the VCFs generated by *vcfsim* align with the theoretical expectations of both nucleotide diversity and F_ST_, with no errors introduced by the simulation or post-processing steps (Figs. 2 and 3). Both tools we employed for computing nucleotide diversity (vcftools, pixy) show identical results (Fig. 2A, B). Missing data (both sites and individual genotypes) are also exactly represented as specified in all output files (Fig. 2C, D). Together, these results validate the accuracy of *vcfsim* in replicating expected genetic diversity and missing data in simulated VCF files.

Our results also demonstrate that *vcfsim* is highly flexible, and capable of generating VCFs with varied ploidy levels and missing data. For example, simulations of tetraploid organisms (Fig. 5B) and diploid organisms with heterogametic sex chromosomes (Fig. 6) accurately reflect theoretical diversity expectations. Patterns of missing data can also be finely tuned to match the properties of a wide variety of organisms (Fig. 7). As such, *vcfsim* will be a useful tool across a wide variety of biological systems.

While a relatively straightforward tool, *vcfsim* fills an important gap in available tools and has many potential applications. One use of *vcfsim* is to benchmark software tools designed for genetic analysis, ensuring that they perform correctly across different configurations of missing data or ploidy. Additionally, *vcfsim* could be used to generate datasets for training different machine learning models, such as neural networks aimed at identifying genetic variants or inferring population structure. These datasets could also be used to conduct power analyses for population genetic inference, helping researchers assess how different levels of missing data or sample sizes affect the ability to detect evolutionary signals or demographic events.

### Limitations

While *vcfsim* provides an accurate and flexible tool for simulating VCFs, there are several important limitations to consider. First, *vcfsim* simulates VCFs with either completely known or completely missing data. In real-world sequencing data, however, we often encounter intermediate cases, such as sites with low sequencing depth or uncertain genotype likelihoods. *vcfsim* does not currently simulate these complexities (but see *vcfgl* [15]). This also includes explicit simulation of sequencing errors, as vcfsim is population genetics-based and does not simulate the sequencing process per se. Another limitation is the size of the all-sites VCFs generated by *vcfsim:* because these files include both variant and invariant sites, they can become quite large, particularly for larger genomes. While the inclusion of invariant sites is essential for certain types of analyses [2], it can create challenges in terms of storage and computational efficiency (which are also true for empirical, non-simulated all-sites VCFs). Handling large all-sites VCFs may require significant memory and processing resources, which can be a bottleneck in large-scale simulations or analyses.

## Conclusions

*Vcfsim* provides a simple, one-step solution for generating all-sites VCFs with customizable missing data and ploidy configurations. By addressing several limitations in current VCF generation workflows, *vcfsim* simplifies the process of creating realistic test datasets for use in genomic research and software validation. The ability to accurately simulate and format genetic variation and introduce controlled amounts of missing data opens new possibilities for benchmarking tools, training machine learning models, and conducting power analyses in population genetics.

### Availability and requirements

- Project name: vcfsim
- Project home page: http://github.com/samuk-lab/vcfsim
- Operating system(s): Platform independent
- Programming language: Python
- Other requirements: Python ≥ 3.6, conda or mamba
- License: MIT
- Restrictions to use by non-academics: No

## Data Availability

The source code for vcfsim is available under an MIT license here: http://github.com/samuk-lab/vcfsim. The code used to perform benchmarking and accuracy validation is available under an MIT license here: https://github.com/samuk-lab/vcfsim-benchmarking-and-test-analysis. All validation datasets used in this study were generated using vcfsim itself; no external empirical datasets were used.

## Abbreviations

VCF: Variant Call Format
SNP: Single nucleotide polymorphism
HMM: Hidden Markov model
